# Grupos de riesgo citogenético de leucemia mieloide aguda pediátrica a partir del análisis de supervivencia en un hospital de referencia para cáncer en Perú

**DOI:** 10.7705/biomedica.5747

**Published:** 2021-06-15

**Authors:** Yésica Llimpe

**Affiliations:** 1 Departamento de Ciencias Dinámicas, Universidad Nacional Mayor de San Marcos, Lima, Perú Universidad Nacional Mayor de San Marcos Departamento de Ciencias Dinámicas Universidad Nacional Mayor de San Marcos Lima Peru

**Keywords:** leucemia mieloide aguda, análisis citogenético, cariotipo, pediatría, supervivencia, aberraciones cromosómicas, Leukemia, myeloid, acute, cytogenetic analysis, karyotype, pediatrics, survival, chromosome aberrations

## Abstract

**Introducción.:**

La leucemia mieloide aguda es una neoplasia heterogénea caracterizada por la proliferación de células mieloides inmaduras. El análisis citogenético ha revelado la presencia de aberraciones cromosómicas de importancia en el pronóstico del paciente.

**Objetivo.:**

Determinar los grupos de riesgo citogenético de pacientes pediátricos con leucemia mieloide aguda a partir de la supervivencia global.

**Materiales y métodos.:**

Se hizo un estudio observacional de corte transversal. Se incluyeron los registros clínicos de los pacientes pediátricos con diagnóstico de leucemia mieloide aguda *de novo* admitidos en el Instituto Nacional de Enfermedades Neoplásicas entre el 2001 y el 2011 y sometidos a análisis citogenético de médula ósea. Los grupos de riesgo citogenético se establecieron según los criterios del *Medical Research Council*. Las curvas de supervivencia global se elaboraron con el método de Kaplan-Meier y se compararon mediante la prueba de Mantel-Cox y una regresión de Cox, utilizando el programa R, versión 3.3.2.

**Resultados.:**

Se incluyeron 130 pacientes, 68 varones (52,3%) y 62 mujeres (47,7%), mayoritariamente del subtipo M2 (33%). La edad promedio fue de 7,7 (rango de 0 a 15 años). Se observaron aberraciones cromosómicas en el 60,8% y la más frecuente fue la traslocación t(8;21). Según el análisis de supervivencia global, se observaron dos grupos de riesgo citogenético: favorable y desfavorable.

**Conclusión.:**

Se determinaron dos grupos de riesgo citogenético: alto (o desfavorable) y estándar (o favorable).

## Abstract (en)

**Introduction:**

Acute myeloid leukemia is a heterogeneous disorder characterized by immature myeloid cell proliferation. Cytogenetic analysis has revealed the presence of chromosomal aberrations important to patient prognosis.

**Objective:**

To determine cytogenetic risk groups of pediatric patients with acute myeloid leukemia according to overall survival.

**Materials and methods:**

In this cross-sectional observational study, the clinical records of pediatric patients diagnosed with *de novo* acute myeloid leukemia admitted to the *Instituto Nacional de Enfermedades Neoplásicas* between 2001 and 2011 with cytogenetic analysis of bone marrow were included. Cytogenetic risk groups were established according to the criteria of the Medical Research Council. Overall survival curves were generated with the Kaplan-Meier method and compared using the Mantel-Cox test and Cox regression with the software R, version 3.3.2.

**Results:**

A total of 130 patients were included, 68 males (52.3%) and 62 females (47.7%), most of them with subtype M2 (33%). The average age was 7.7 years (range: 0-15 years).

Chromosomal aberrations were observed in 60.8% of the patients, the most frequent of which was the translocation t(8;21). According to the overall survival analysis, two cytogenetic risk groups were established: favorable and unfavorable.

**Conclusion:**

Two groups of cytogenetic risk were determined: high (or unfavorable) and standard (favorable).

La denominación *leucemia mieloide aguda* agrupa un conjunto heterogéneo de enfermedades malignas caracterizadas por la proliferación descontrolada de las células progenitoras mieloides de la médula ósea ^1^^,^^2^, la cual se diagnostica con mayor frecuencia en personas entre los 65 y los 74 años (edad promedio, 68) ^3^. En los niños tiene una incidencia de 7 casos por cada millón de menores de 15 años ^4^, siendo más alta entre los 0 y los 4 años ^5^. Esta condición representa del 15 al 25% de las leucemias agudas de la infancia ^1^ y ya está superando a la leucemia linfoblástica aguda como la causa principal de mortalidad infantil por leucemia ^6^.

La clasificación franco-anglo-estadounidense (*French-American-British*, FAB) de la leucemia mieloide aguda se basó en criterios morfológicos y citoquímicos ^7^ y estableció ocho subtipos (M0 a M7) ^2^. Posteriormente, la Organización Mundial de la Salud (OMS) estableció una nueva clasificación de las neoplasias mieloides, incorporando las aberraciones cromosómicas recurrentes ^8^. A partir de dicha clasificación, se han caracterizado traslocaciones cromosómicas como la t(8;21) o la t(15;17), las cuales resultan en la formación de proteínas quiméricas (RUNX1-RUNX1T1 y PML-RARA, respectivamente) que alteran el proceso de la maduración normal de las células precursoras mieloides. Asimismo, se han detectado cambios a nivel molecular implicados en el desarrollo de la leucemia mieloide aguda ^2^.

En el 70 al 80% de los casos de leucemia mieloide aguda en niños, se presentan aberraciones cromosómicas ^9^^-^^11^. Las más frecuentes son la t(8;21)(q22;q22) y la inv(16)(p13q22) que, en conjunto, se denominan *Core Binding Factor AML* (CBF-AML), y además, la t(15;17)(q22;q21) y los reordenamientos de la 11q23/MLL ^4^.

Con base en los hallazgos citogenéticos, se pueden establecer grupos de pronóstico o de riesgo ^12^. Equipos internacionales de investigación como el *Southwest Oncology Group* (SWOG), el *Cancer and Leukemia Group B* (CALGB) y el *Medical Research Council* (MRC), han propuesto una clasificación citogenética orientada al pronóstico. Es importante señalar que las clasificaciones en grupos de riesgo citogenético se basan en los estudios que incluyeron niños y adultos, en los cuales predominaron los menores de 60 años ^13^. Las evidencias actuales sugieren que, aunque la leucemia mieloide aguda de los niños y la de los adultos tienen fenotipos comunes, representarían enfermedades genéticas distintas, con reordenamientos o rearreglos cromosómicos que probablemente constituyen el evento inicial de gran parte del desarrollo de la leucemia infantil ^14^.

Los datos publicados recientemente por el proyecto *Therapeutically Applicable Research to Generate Effective Treatments* (TARGET), un esfuerzo colaborativo para caracterizar mutaciones y aspectos relacionados a procesos de transcripción y epigenética de la leucemia mieloide aguda pediátrica, han ampliado significativamente la comprensión de su biología y de la forma en que difiere de la de los adultos ^15^. A diferencia de la acumulación gradual de cambios genéticos de esta condición en los adultos, en los niños, las traslocaciones cromosómicas son a menudo las responsables del desarrollo de la enfermedad. Por sí misma, ya la leucemia mieloide aguda pediátrica comprende una colección de enfermedades molecularmente diversas con fenotipos similares ^6^ y parece constituir un subgrupo específico con características clínicas y biológicas propias ^16^, por lo que es probable que ninguna estrategia de tratamiento único sea efectiva para todos los subtipos de la enfermedad ^6^.

El reto de la determinación de blancos terapéuticos en la leucemia mieloide aguda ha sido su heterogénea composición genética y molecular, que frecuentemente evoluciona en variantes génicas que impulsan el inicio y la progresión de la enfermedad ^15^. Aunque ha habido mejoras en su tratamiento, solo alrededor del 60% de los pacientes logra una supervivencia a largo plazo y hasta el 10% de los niños con la enfermedad muere por complicaciones directas del tratamiento ^6^.

En contraste con los avances en la comprensión de los eventos moleculares que ocurren en la leucemia mieloide aguda pediátrica a partir del análisis de la expresión génica ^17^^,^^18^ y de la secuenciación ^19^, aún no se usan tratamientos específicos, con excepción de los inhibidores de la cinasa de la tirosina activada por FLT3, empleados en el marco de investigaciones ^6^.

A diferencia de los estudios moleculares, el análisis citogenético tiene la ventaja de ofrecer un panorama completo de todo el genoma nuclear y, aunque otros factores, como los de tipo clínico, juegan un papel importante en el tratamiento, los cambios citogenéticos constituyen el factor de pronóstico más sólido para la evaluación de la remisión completa y la supervivencia global en la leucemia mieloide aguda ^2^; además, proporcionan un esquema para el tratamiento estratificado según el riesgo ^20^.

Dicha estrategia debería ser adoptada por todas las instituciones de salud del país a cargo de pacientes con leucemia mieloide aguda e incluirse en sus guías de prácticas clínicas, como lo han hecho el Instituto Nacional de Enfermedades Neoplásicas y el Instituto Regional de Enfermedades Neoplásicas.

No se encontraron estudios en Perú que evaluaran los objetivos del presente estudio, es decir, la supervivencia global y los grupos de riesgo a partir de los hallazgos citogenéticos en pacientes pediátricos con leucemia mieloide aguda.

## Materiales y métodos

Se llevó a cabo un estudio observacional descriptivo en el Laboratorio de Genética del Instituto Nacional de Enfermedades Neoplásicas en pacientes menores de 15 años de edad con diagnóstico de leucemia mieloide aguda *de novo*, admitidos entre el 2001 y el 2011, a quienes se les había hecho el estudio citogenético de médula ósea en el momento del diagnóstico y en las metafases analizables por citogenética convencional. Los cariotipos se revisaron y describieron según la *International System for Human Cytogenetics Nomenclature.* La supervivencia global se consideró como el tiempo desde el ingreso hasta la muerte de los pacientes y, para evaluarla a 5 años, se utilizó el método de Kaplan-Meier ^21^^,^^22^. Los sujetos perdidos durante el seguimiento se censuraron en la fecha del último contacto registrado.

Los grupos de riesgo citogenético se establecieron siguiendo los criterios del *Medical Research Council* modificados por Harrison, *et al.*^10^ (cuadro 1) en un estudio anterior, en el que incluyeron casos con características semejantes a las del presente estudio. Los grupos de riesgo citogenético establecidos por dicho consejo son: “alto” (o desfavorable), “intermedio” y “bajo” (o favorable). Las curvas de supervivencia se compararon mediante la prueba de Mantel-Cox (*log-rank test*). Según estos hallazgos, se determinaron grupos de riesgo citogenético y se analizaron mediante una regresión de Cox utilizando el programa R, versión 3.3.2.

**Cuadro 1:** Clasificación del riesgo citogenético según el *Medical Research Council*

Grupo de riesgo citogenético	Hallazgo
Riesgo bajo	inv(16)(p13q22)/t(16;16)/del(16q), t(15;17)(q24;q21), t(8;21) (q22;q22) con aberraciones secundarias o sin ellas
Riesgo intermedio	Aberraciones no clasificadas como de riesgo alto o bajo
Riesgo alto	Aberraciones del 3q [excluyendo t(3;5)(q21~25;q31~35)], inv(3)(q21q26)/t(3;3)(q21;q26), add(5q), del(5q), -5, add(7q)/ del(7q), -7, t(6;11)(q27;q23), t(10;11)(p11~13;q23), t(11q23) [con exclusión de t(9;11)(p21~22;q23) y t(11;19)(q23;p13)], t(9;22)(q34;q11), -17/aberraciones de 17p, cariotipo complejo (≥4 aberraciones no relacionadas)

### Consideraciones éticas

En ningún momento hubo intervención en los pacientes. Los datos del análisis citogenético se obtuvieron del registro en las historias clínicas, por lo que no se requirió del consentimiento informado de los pacientes. El proyecto fue revisado y aprobado por el Comité Revisor de Protocolos del Departamento de Investigación del Instituto Nacional de Enfermedades Neoplásicas, código 14-89.

## Resultados

### Distribución por subtipo, edad y sexo

En el periodo de 10 años -de 2001 a 2011-, se encontraron 328 registros de pacientes pediátricos con diagnóstico de leucemia mieloide aguda, de los cuales 130 cumplían con los criterios de inclusión. Los casos excluidos presentaban datos incompletos en la historia clínica, no tenían estudio citogenético o la morfología cromosómica no era la adecuada para el estudio citogenético.

La distribución por sexo fue de 1,1 a 1 (masculino, n=68; femenino, n=62). La edad promedio de los pacientes fue de 7,7 años (rango de 0 a 15), y se observaron dos picos de distribución de casos: entre 1 y 2 años (n=22), y entre 9 y 10 años (n=24). Según la clasificación FAB, los pacientes con leucemia mieloide aguda eran mayoritariamente del subtipo M2 (n=43, 33%), seguidos, en orden, por el M3 (n=8), el M4 (n=5), el M0 (n=3), el M1 (n=3), el M5 (n=3), el M6 (n=3) y el M7 (n=1) (33%), en tanto que 61 casos no se clasificaron en ningún subtipo específico.

### Aberraciones cromosómicas

Las metafases se analizaron mediante la técnica de citogenética convencional (bandas G). El 60,8% de los casos incluidos (n=79) presentaba alguna aberración cromosómica numérica o estructural adquirida. Se observó la trisomía 21 (aberración cromosómica constitucional) en cinco pacientes con síndrome Down con edades entre 1 y 9 años, tres de ellos con aberraciones cromosómicas adicionales.

La aberración cromosómica adquirida más frecuente fue la traslocación t(8;21)(q22;q22), observada en el 35,4% de los casos analizados (46/130) y en el 58,2% de los casos con aberraciones cromosómicas (46/79). Se encontró también pérdida del cromosoma sexual (-X o -Y) en 23 casos (17,7%), mayoritariamente en aquellos con la traslocación t(8;21).

Las aberraciones que siguieron en frecuencia fueron la t(15;17)(q24;q21) (3,1%), la t(9;11)(p22;q23) (1,5%), la inv(16)(q22) (0,8%), la t(6;8)(q25;q22) (0,8%), la t(6;9)(p23;q34) (0,8%) y la t(9;22)(q34;q11) (0,8%). Otras aberraciones cromosómicas incluyeron monosomías, trisomías, deleciones, adiciones, cromosomas marcadores y en anillo (cuadro 2).

**Cuadro 2:** Aberraciones cromosómicas más frecuentes

Subtipo de leucemia mieloide aguda	Descripción	Aberraciones cromosómicas más frecuentes*
M0	Leucemia mieloide aguda con maduración mínima	-
M1	Leucemia mieloide aguda sin maduración	-
M2	Leucemia mieloide aguda con maduración	t(8;21)(q22;q22), del(7q), -X, -Y, +4, +8, +mar t(15;17)(q24;q21)
M3	Leucemia promielocítica aguda	
M4	Leucemia mielomonocítica aguda	-
M5	Leucemia monocítica aguda	-
M6	Leucemia eritroide aguda	-
M7	Leucemia megacarioblástica aguda	-

*Consideradas a partir de dos casos

### Comparación de las curvas de supervivencia entre los grupos de riesgo citogenético

**Figura 1:**
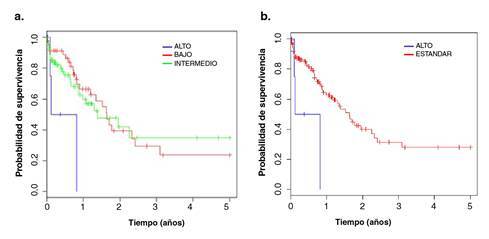
Curvas de supervivencia global de los grupos de riesgo citogenético: “alto”, “bajo” e “intermedio” según el *Medical Research Council* modificado por Harrison, *et al*., **(a)** y “alto” y “estándar” (clasificación según la propuesta) **(b)**, a partir de la población de pacientes pediátricos con diagnóstico de leucemia mieloide aguda admitidos en el Instituto Nacional de Enfermedades Neoplásicas durante el periodo 2001-2011

El riesgo no se incrementó al presentarse alguna aberración cromosómica, en comparación con los casos con cariotipo normal (p=0,375). Al clasificar los hallazgos citogenéticos según los criterios del *Medical Research Council* modificados por Harrison, *et al.*^10^, se trazaron tres curvas de supervivencia (figura 1a) que, según la prueba de Mantel-Cox, no mostrarían diferencia significativa (p=0,0562) (cuadro 3). A partir de las curvas de supervivencia obtenidas, se propuso la siguiente clasificación de grupos de riesgo citogenético: “estándar” y “alto” (figura 1b), que corresponden a riesgo favorable y desfavorable, respectivamente. La prueba de Mantel-Cox indicó que sí hubo diferencia significativa entre los grupos de riesgo “estándar” y “alto” (p=0,0171) (cuadro 3).

**Cuadro 3:** Prueba de Mantel-Cox para grupos de riesgo citogenético según el *Medical Research Council* modificado por Harrison, et al., y según la presente propuesta

Grupos de riesgo citogenético			
MRC modificado		Propuesta	
Bajo	n=7	Estándar	n=13
Intermedio	n=66	Alto	n=6
Alto	n=6		
P=0,0562		P=0,0171	

*MRC: Medical Research Council*

**Figura 2:**
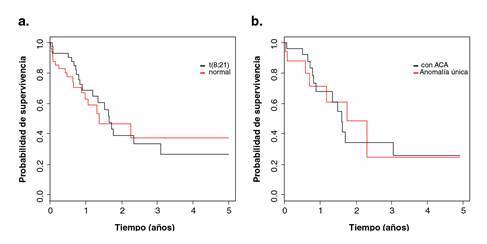
Curvas de supervivencia global de los grupos con la traslocación t(8;21) y con cariotipo normal (a) y de los grupos con traslocación t(8;21) como anomalía única más aberraciones cromosómicas adicionales (b); a partir de la población de pacientes pediátricos con diagnóstico de leucemia mieloide aguda admitidos en el Instituto Nacional de Enfermedades Neoplásicas durante el periodo 2001-2011

El mismo análisis se realizó entre los grupos con t(8;21) y con cariotipo normal (p=0,694) (figura 2a), así como entre los grupos con t(8;21) como anomalía única y con la misma anomalía más aberraciones cromosómicas adicionales (p=0,995) (figura 2b) (cuadro 4).

**Cuadro 4.** Prueba de Mantel-Cox para grupos con traslocación t(8;21), con aberraciones cromosómicas adicionales y con cariotipo normal

Grupos de riesgo citogenético			
t(8;21)	n=46	t(8;21) como anomalía única	n=18
Cariotipo normal	n=51	t(8;21) con aberraciones cromosómicas adicionales	n=28
p = 0,694		p = 0,995	

Mediante un análisis univariado por regresión de Cox para el factor de grupo de riesgo citogenético según la propuesta, se obtuvo un cociente de riesgo (*hazard ratio,* HR) de 0,1698 (con el grupo “estándar” como referencia), con un valor de p=0,00113 (cuadro 5).

**Cuadro 5:** Análisis univariado mediante regresión de Cox para el factor grupo de riesgo citogenético

	Hazard ratio		IC95%		p
	Exp(coef)	Exp(-coef)	Inferior	Superior	
Grupo de riesgo (estándar)	0,1698	5,888	0,05839	0,4939	0,00113

## Discusión

Los estudios genéticos sobre leucemia mieloide aguda generalmente incluyen un número limitado de pacientes pediátricos ^11^^,^^23^^,^^24^. La mayoría de los estudios sólidos sobre la enfermedad en niños son de tipo multicéntrico ^5^^,^^6^^,^^25^^,^^26^, en tanto que el presente estudio incluyó pacientes registrados en una sola institución de referencia especializada en cáncer a nivel nacional en Perú.

### Distribución por subtipo, edad y sexo

Según la clasificación FAB, el subtipo más frecuente fue el M2, observado igualmente en Brasil ^27^ y en los Estados Unidos ^28^^,^^29^, pero no así en Líbano y en Italia, donde la mayoría fueron del subtipo M3 y M5, respectivamente ^30^^,^^31^. En los reportes que incluyen todos los grupos etarios, los subtipos mayoritarios fueron el M2, en los Estados Unidos, Pakistán y Alemania ^32^^-^^34^; el M3 en Sudán y Brasil ^35^^,^^36^; el M4 en Pakistán ^37^, y el M5 y el M0 en Turquía ^1^. Esto evidencia las diferencias geográficas en la distribución de los subtipos predominantes, posiblemente debidas a factores étnicos y ambientales ^35^. Lo mismo ocurrió con las distribuciones por edad y sexo.

### Aberraciones cromosómicas

Los datos sobre la distribución de los cambios cromosómicos en la leucemia mieloide aguda pediátrica son escasos y provienen fundamentalmente de lo observado en pacientes adultos ^10^. El presente estudio evidenció que el 60,8% de los pacientes menores de 15 años con la enfermedad presentaba aberraciones cromosómicas; en otros reportes se han encontrado porcentajes mayores en niños y adolescentes, entre el 67 y el 80% ^10^^,^^30^^,^^38^.

Esta discrepancia podría deberse a los diversos grados de experiencia de los analistas, así como al tamaño de las aberraciones cromosómicas, ya que algunas pueden ser sutiles, como la inv(16)(p13q22), la t(15;17) (q24;q21) y la t(11;19)(q23;p13.1) (39). Asimismo, la detección mejora con la utilización conjunta de la citogenética convencional y otras técnicas como la hibridación *in situ* con fluorescencia (*Fluorescent in situ Hybridization,* FISH) y la reacción en cadena de la polimerasa (PCR). Se recomienda que la tamización inicial para la detección de aberraciones cromosómicas asociadas con cáncer se haga con la citogenética convencional para, después, emplear un procedimiento de FISH apropiado ^40^. Por otro lado, el uso de la RT-PCR complementa el análisis por FISH en la detección de múltiples genes de fusión asociados con la leucemia mieloide aguda ^41^.

La t(8;21)(q22;q22) fue la aberración cromosómica adquirida más frecuente, con el 35,4% de los casos analizados. Se han reportado porcentajes menores ^42^^,^^43^ y similares en otros estudios ^44^, lo que refleja la heterogeneidad geográfica de las aberraciones cromosómicas en las neoplasias ^45^. En el 45,7% de los casos con t(8;21)(21/46), había también pérdida del cromosoma sexual, la mayoría en pacientes de sexo masculino (15/21), semejante a lo observado en pacientes adultos ^46^^,^^47^.

Otras aberraciones cromosómicas con pronóstico favorable, como la t(15;17)(q24;q21), solo se observaron en cuatro casos, la inv(16)(p13q22) en un caso, y la t(9;22)(q34;q11) y la t(6;9)(p23;q34) en un caso cada una, ambas con pronóstico no favorable.

### Análisis y comparación de las curvas de supervivencia

El análisis citogenético se considera uno de los factores pronósticos más valiosos en la leucemia mieloide aguda pediátrica ^48^. En este sentido, en este estudio se buscó determinar los grupos de riesgo a partir de los hallazgos citogenéticos, considerando la supervivencia global a los cinco años. Las aberraciones cromosómicas encontradas se agruparon según los criterios del *Medical Research Council* modificados por Harrison, *et al.*^10^, los cuales excluyen los casos de leucemia promielocítica y a los pacientes con síndrome Down; en estos últimos, la trisomía 21 constitucional ha demostrado tener un impacto en el resultado del tratamiento de la leucemia mieloide aguda ^49^. Los criterios de dicho consejo están entre los más estandarizados para la clasificación de pacientes en grupos de riesgo citogenético ^50^ y fueron empleados por Harrison, *et al.*^10^, en uno de los estudios con mayor número de pacientes pediátricos con leucemia mieloide aguda.

En el presente estudio, al comparar las curvas de supervivencia global de los pacientes con alteración cromosómica adquirida y aquellos con cariotipo normal, se determinó que la sola presencia de una aberración cromosómica no incrementaba el riesgo en ellos. Al comparar las curvas de los grupos de riesgo citogenético según el *Medical Research Council* modificado por Harrison, *et al.*^10^, tampoco se encontraron diferencias significativas; la gráfica reflejó, más bien, una acentuada diferencia en la curva correspondiente al grupo de “riesgo alto” frente a las otras dos curvas ( figura 1a).

A partir de esos hallazgos, se establecieron dos grupos de riesgo citogenético: de “riesgo alto” y de “riesgo estándar”, en tanto que los grupos “intermedio” y “bajo” conformaron un único grupo dado que sus curvas de supervivencia presentaron una superposición muy cercana.

Una comprobación de esta propuesta resulta de la comparación de las curvas de supervivencia de los grupos de riesgo conformados por pacientes con cariotipo normal frente a aquellos con la t(8;21). Según los criterios de Harrison, *et al.*^10^, el cariotipo normal se asigna al grupo de “riesgo intermedio” y, la t(8;21), al grupo de “riesgo bajo”. Sin embargo, no se encontró una diferencia significativa al analizar las curvas de supervivencia de los pacientes con cariotipo normal frente a las de quienes presentaron la t(8;21); por lo tanto, los pacientes con la t(8;21) tendrían un pronóstico semejante a aquellos con cariotipo normal y podrían incluirse en el mismo grupo de riesgo citogenético.

El valor pronóstico de la t(8;21) fue independiente de la presencia de aberraciones cromosómicas adicionales en la misma clona, ya que al comparar las curvas de supervivencia de pacientes con la t(8;21) como anomalía única y aquellos con la t(8;21) más aberraciones adicionales, no se encontró una diferencia significativa. Se concluyó, por lo tanto, que el cariotipo normal y la t(8;21), acompañada o no de otras aberraciones, tenían el mismo valor pronóstico.

Cuando se elaboraron las curvas de supervivencia con los dos grupos de riesgo citogenético propuestos, la prueba de Mantel-Cox evidenció una diferencia significativa entre ambos, en tanto que, en el análisis univariado con la regresión de Cox, se registró un cociente de riesgo de 0,1698, es decir, hubo una diferencia en la supervivencia global entre el grupo de “riesgo alto” y el de “riesgo estándar”.

Todos los pacientes del grupo de “riesgo alto” tenían 2 años de edad y presentaban la t(6;9)(p23;q34), la t(9;22)(q34;q11), la del(12)(p11.2), la del(5q), la -7, -10, la t(8;12)(q22;q24), la del(10)(q13.2) y cromosomas marcadores. Las cinco primeras están dentro del grupo de “riesgo alto” según Harrison, *et al*. ^10^, y se han relacionado con el mal pronóstico en varios estudios ^51^^-^^55^. En cuanto a las aberraciones cromosómicas recurrentes raras, hay poco consenso sobre el pronóstico, además de que su incidencia es menor de 2% y podrían dar cuenta del 10%, aproximadamente, de las leucemias mieloides agudas consideradas como de pronóstico intermedio o adverso ^56^. Así, la t(8;12)(q22;q24), clasificada en el grupo de “riesgo alto” es rara, lo mismo que la t(6;8)(q25;q22), la del(11)(q14) y la t(6;13)(q23;q22), cuyo valor pronóstico no se ha podido determinar debido al número reducido de casos.

Otra limitación fue el no disponer de la herramienta citogenética molecular, que hubiera permitido el análisis de aquellos casos con metafases de pobre morfología cromosómica, ya se logra la identificación de genes de fusión, incluso en células en interfase. Tampoco se disponía del análisis genético molecular, el cual no se hizo en el momento de la admisión de los pacientes porque su establecimiento como prueba de rutina fue posterior.

Los estudios recientes sobre la supervivencia de los pacientes con neoplasias hematológicas incluyen datos de tipo molecular, entre ellos, la identificación de genes de fusión formados a partir de aberraciones cromosómicas recurrentes, además de marcadores moleculares que incorporan mutaciones en los genes *FLT3*, *NPM1*, *WT1*, *PTPN11* y *CEPBA*^44^, y que constituyen variables de análisis en la leucemia mieloide aguda pediátrica.

Otra variable por considerar es el tipo de tratamiento, pues una aberración que confiere un pronóstico adverso, tratada con un determinado esquema terapéutico, podría cambiar cuando se usa uno distinto ^39^. En efecto, la optimización de la estrategia de inducción y posterior a la remisión, una mejor terapia de soporte, el amplio uso del trasplante alogénico de células madre hematopoyéticas en pacientes de alto riesgo y la contribución de la estratificación en grupos de terapia dirigida, han aumentado la supervivencia de los pacientes pediátricos con la enfermedad ^57^. En el presente estudio, los pacientes recibieron tratamiento según la guía de práctica clínica de leucemia mieloide aguda del Instituto Nacional de Enfermedades Neoplásicas (estudio clínico o combinación de citarabina y antraciclínicos o antracenedionas).

Se concluyó que había dos grupos de riesgo citogenético en los pacientes pediátricos analizados: un grupo con reacción favorable al tratamiento (“riesgo estándar”) y otro con reacción desfavorable (“riesgo alto”), hallazgo que simplificaría el manejo clínico de los pacientes. El análisis citogenético convencional, entonces, debe aplicarse como prueba inicial y complementarse con el análisis citogenético molecular o genético molecular. Si bien la proporción de casos de leucemia mieloide aguda es mayor en adultos que en niños, su manejo en ellos se ha basado en los datos obtenidos de pacientes adultos ^58^, por lo que deben orientarse esfuerzos hacia este grupo de pacientes cuyo perfil es distinto.
